# Tetrahydroxy stilbene glucoside alleviates palmitic acid-induced inflammation and apoptosis in cardiomyocytes by regulating miR-129-3p/Smad3 signaling

**DOI:** 10.1186/s11658-018-0125-x

**Published:** 2019-02-19

**Authors:** Yong ZOU, Min KONG

**Affiliations:** 1 Department of Cardiovascular Medicine, Wuhan No. 6 Hospital, Hospital Affiliated to Jianghan University, No. 168, Xianggan Road, Wuhan, 430016 People’s Republic of China; 2 Department of Pharmacy, Wuhan No. 6 Hospital, Hospital Affiliated to Jianghan University, No. 168, Xianggan Road, Wuhan, 430016 People’s Republic of China

**Keywords:** Tetrahydroxy stilbene glucoside, miR-129-3p, Smad3, Cardiomyocytes, Inflammation, Apoptosis

## Abstract

**Objective:**

Tetrahydroxy stilbene glucoside (TSG) has been reported to exert a cytoprotective effect against various toxicants. However, the function and mechanism of TSG in palmitic acid (PA)-induced inflammation and apoptosis in cardiomyocytes are still unknown. The present study was designed to investigate the post-transcriptional mechanism in TSG-treated cardiomyocytes’ inflammation and apoptosis induced by PA.

**Methods:**

The mRNA and protein levels were assayed by reverse transcription-quantitative polymerase chain reaction (RT-qPCR) and western blotting, respectively. The targeted genes were predicted by a bioinformatics algorithm and confirmed by a dual luciferase reporter assay. Cell proliferation was analyzed by CCK-8 assay. Annexin V-fluorescein isothiocyanate/polyimide (annexin V-FITC/PI) staining was used to evaluate apoptosis using flow cytometry.

**Results:**

TSG restricted the detrimental effects, including the activated inflammatory response and apoptosis, of PA in cardiomyocytes, as well as the up-regulation of miR-129-3p and down-regulation of p-Smad3 expression. In addition, bioinformatics and experimental analysis suggested that Smad3 was a direct target of miR-129-3p, which could inhibit or enhance the expression of p-Smad by transfection with miR-129-3p mimics or inhibitors, respectively. Furthermore, our results demonstrated that overexpression of Smad3 reversed the inhibition of inflammation and apoptosis by overexpression of miR-129-3p in PA-stimulated cardiomyocytes.

**Conclusion:**

TSG targeted to miR-129-3p/Smad3 signaling inhibited PA-induced inflammation and apoptosis in cardiomyocytes.

## Introduction

Elevated levels of saturated palmitic acids (PA) in plasma have been proposed as a potential pathogenic factor to induce lipotoxic cardiomyopathy by over-activating inflammation and apoptosis in cardiomyocytes [1, 2]. Previous studies have found that high fat diet (HFD)-induced low grade chronic inflammation, also called metabolic inflammation, which is due to secretion of pro-inflammatory cytokines from macrophages, has a detrimental effect on cardiovascular function, including myocarditis, cardiac hypertrophy and heart failure [3, 4]. In addition, inflammation is implicated in HFD-induced cardiomyocytes’ apoptosis by tumor necrosis factor α (TNF-α)-mediated nuclear factor-kappa B (NF-κB) signaling [5]. Therefore, inhibition of inflammation will provide a new insight into the treatment of cardiomyocyte injury.

Tetrahydroxy stilbene glucoside (2, 3, 5, 4′-tetrahydroxy stilbene-2-O-*β*-D-glucoside; TSG) as the main active ingredient of Heshouwu (*Radix Polygoni Multiflori*), which is a widely used Chinese herbal medicine, is frequently reported to perform cytoprotective effects, including anti-inflammation, anti-oxidative and anti-apoptosis [6–8]. However, the cytoprotective effects and underlying molecular mechanisms of TSG in PA-induced inflammation and apoptosis in H9C2 cardiomyocytes have not been completely clarified.

Mothers against decapentaplegic homolog 3 (also known as Smad family member 3; Smad3) is an important mediator for transforming growth factor β (TGF-β)/Smads signaling, which regulates cell proliferation, differentiation and death [9]. The improvement of Smad3 phosphorylation enhances NF-κB inhibitor alpha (IκBα) degradation and activates NF-κB signaling, which can promote the inflammatory reaction [10]. Moreover, phosphorylated Smad3 combined with phosphorylated Smad2 binds to Smad4, which translocates into the nucleus and can regulate the transcription of several target genes, such as Bax and caspase-3, and induce apoptosis [11, 12].

In the present study, a post-transcriptional regulatory mechanism by miR-129-3p, a noncoding, short, single-stranded, approximately 22 nucleotide RNA [13], was found to regulate PA-induced inflammation and apoptosis in H9c2 cells by targeting inhibition of p-Smad3. We also found that TSG had a protective effect against PA-induced cardiomyocyte dysfunction by regulating the miR-129-3p/Smad3 signaling pathway.

## Materials and methods

### Cell culture

H9c2 cells were obtained from the American Type Culture Collection (ATCC, Bethesda, MD, USA) and were cultured in Dulbecco’s modified Eagle’s medium (DMEM; Gibco; Thermo Fisher Scientific, Inc., Waltham, MA, USA), which contained 10% fetal calf serum (Gibco; Thermo Fisher Scientific, Inc.), 10% L-glutamine, 0.5% penicillin/streptomycin, 10% nonessential amino acids and 10% pyruvate, in a 5% CO_2_ atmosphere at 37 °C. H9C2 cells were treated with PA or combined with TSG (HPLC ≥98%; National Institute for the Control of Pharmaceutical and Biological Products, Beijing, China). All of the experiments were performed in triplicate. PA was purchased from Sigma-Aldrich (cat. no. P5585; Merck KgaA, Germany), and fatty acid free bovine serum albumin (BSA; cat. no. 03117057001; Roche, Basel, Switzerland) was used to increase the solubility of PA. Endotoxin in BSA was neutralized with polymyxin B sulfate (cat. no. 5291; Sigma-Aldrich, Merck KGaA, Germany).

### Cell transfection and plasmid constructs

Pre-miR-Con, pre-miR-129-3p, anti-miR-Con and anti-miR-129-3p were synthesized by RiboBio (Guangzhou, China). H9c2 cells were seeded in 6-well plates and transfected with pre-miR-Con, pre-miR-129-3p, anti-miR-Con and anti-miR-129-3p using Lipofectamine 2000 (Invitrogen; Thermo Fisher Scientific, Inc., Waltham, MA, USA) for 48 h at 37 °C according to the manufacturer’s protocol.

A mammalian expression plasmid (pReceiver-M02-ERBB3; GeneCopoeia, Germantown, MD, USA) was designed to specially express the full-length open reading frame (ORF) of rat Smad3 without miR-129-3p responsive 3′-UTR. An empty plasmid served as a negative control. Overexpressed Smad3 plasmids (vector-AGTRAP) and control plasmids (vector-Con) were transfected into H9c2 cells using Lipofectamine 2000 (Invitrogen; Thermo Fisher Scientific, Inc., Waltham, MA, USA) for 48 h at 37 °C according to the manufacturer’s protocol.

### Cell viability

The proliferation of H9c2 cells was monitored using a CCK-8 assay kit (Dojindo, Japan). The absorbance was measured at 450 nm using a SpectraMax M5 plate reader (Molecular Devices, USA).

### Apoptosis assay

H9c2 cells were incubated with different conditions for 48 h. Annexin V-FITC/PI kit (Becton, Dickinson and Company, New Jersey, USA) was used to stain cells for 15 min, and then a cell apoptosis assay was performed by flow cytometry assay (FACScan, BD Biosciences, San Jose, CA, USA) and analyzed by CELL Quest 3.0 software (BD Biosciences).

### Measurement of inflammatory cytokines

The levels of tumor necrosis factor-α (TNF-α), interleukin (IL)-1β and IL-6 in cellular supernatant were detected using the bioactive ELISA assay (Elabscience Biotechnology Co., Ltd., Wuhan, China) with a SpectraMax M5 ELISA plate reader (Molecular Devices, LLC, Sunnyvale, CA, USA), according to the manufacturer’s protocol.

### Luciferase reporter assay

The sequence of miR-129-3p was obtained using online prediction software and synthesized by RiboBio (Guangzhou, China). The wild-type (WT) or mutant-type (MUT) 3′-UTR of Smad3 was inserted into the multiple cloning sites of the luciferase expressing pMIR-REPORT vector (Ambion; Thermo Fisher Scientific, Inc.). For the luciferase assay, H9c2 cells (1 × 10^5^) were seeded into 24 wells and co-transfected with luciferase reporter vectors containing the WT or MUT 3′-UTR of Smad3 (0.5 μg) combined with pre-miR-Con or pre-miR-129-3p (100 nM) using Lipofectamine 2000 (Invitrogen; Thermo Fisher Scientific, Inc.), according to the manufacturer’s protocol. The luciferase activity was measured using a luciferase reporter assay kit (Promega, Madison, WI, USA) according to the manufacturer’s protocol.

### Reverse transcription-quantitative polymerase chain reaction (RT-qPCR)

Total RNA was extracted using TRIzol (Invitrogen; Thermo Fisher Scientific, Inc., Waltham, MA, USA), according to the manufacturer’s protocol. miR-129-3p was detected using the TaqMan MicroRNA assay (Applied Biosystems, Foster City, USA) following the manufacturer’s instructions. U6 snRNA was used as an endogenous control. In addition, 2 μg of total RNA was used to synthesize cDNA with Moloney murine leukemia virus reverse transcriptase (Invitrogen; Thermo Fisher Scientific, Inc.). RT-qPCR was performed by the Applied Biosystems 7300 Real-Time PCR System (Thermo Fisher Scientific, Inc.) with the TaqMan Universal PCR Master Mix (Thermo Fisher Scientific, Inc.). The relative expression levels of mRNA were calculated using the 2^-ΔΔCt^ method [14] and normalized to glyceraldehyde 3-phosphate dehydrogenase (GAPDH). The primers of Smad3, TNF-α, IL-1β, IL-6 and GAPDH were synthesized by Invitrogen (Invitrogen, Shanghai, China).

### Western blotting

Proteins were extracted with radioimmunoprecipitation assay (RIPA) buffer (cat. no: P0013B; Beyotime Institute of Biotechnology, Haimen, China). The concentrations were determined using the Bicinchoninic Acid Kit for Protein Determination (cat. no: BCA1-1KT; Sigma-Aldrich; Merck KGaA). Western blotting assay was performed as previously described [7]. Smad3 (cat. no: 9513, dilution: 1: 1000), p-Smad3 (cat. no: 9520, dilution: 1: 1000) and NF-κB/p65 (cat. no: 6956, dilution: 1: 500) were purchased from Cell Signaling Technology. Following three washes with TBST, the membranes were incubated with the appropriate horseradish peroxidase-conjugated secondary antibody (cat. no: sc-516,102; dilution: 1:10,000; Santa Cruz Biotechnology) at room temperature for 2 h and visualized by chemiluminescence (Thermo Fisher Scientific, Inc.). Signals were analyzed with Quantity One software version 4.5 (Bio Rad Laboratories, Inc., Hercules, CA, USA). β-actin (cat. no. sc-130,065; 1: 2000; Santa Cruz Biotechnology) and histone (cat. no: 9715; dilution: 1: 2000; Cell Signaling Technology, Inc., USA) were used as the control antibodies.

### Statistical analysis

Data were presented as mean ± SD. Statistical analysis was performed using GraphPad Prism Version 7.0 (GraphPad Software, Inc., La Jolla, CA, USA). Inter-group differences were analyzed by one-way analysis of variance (ANOVA), followed by Tukey’s post hoc analysis. *P* less than 0.05 was considered to indicate a statistically significant difference.

## Results

### TSG prevented PA-induced apoptosis in H9c2 cardiomyocytes

**Fig. 1:**
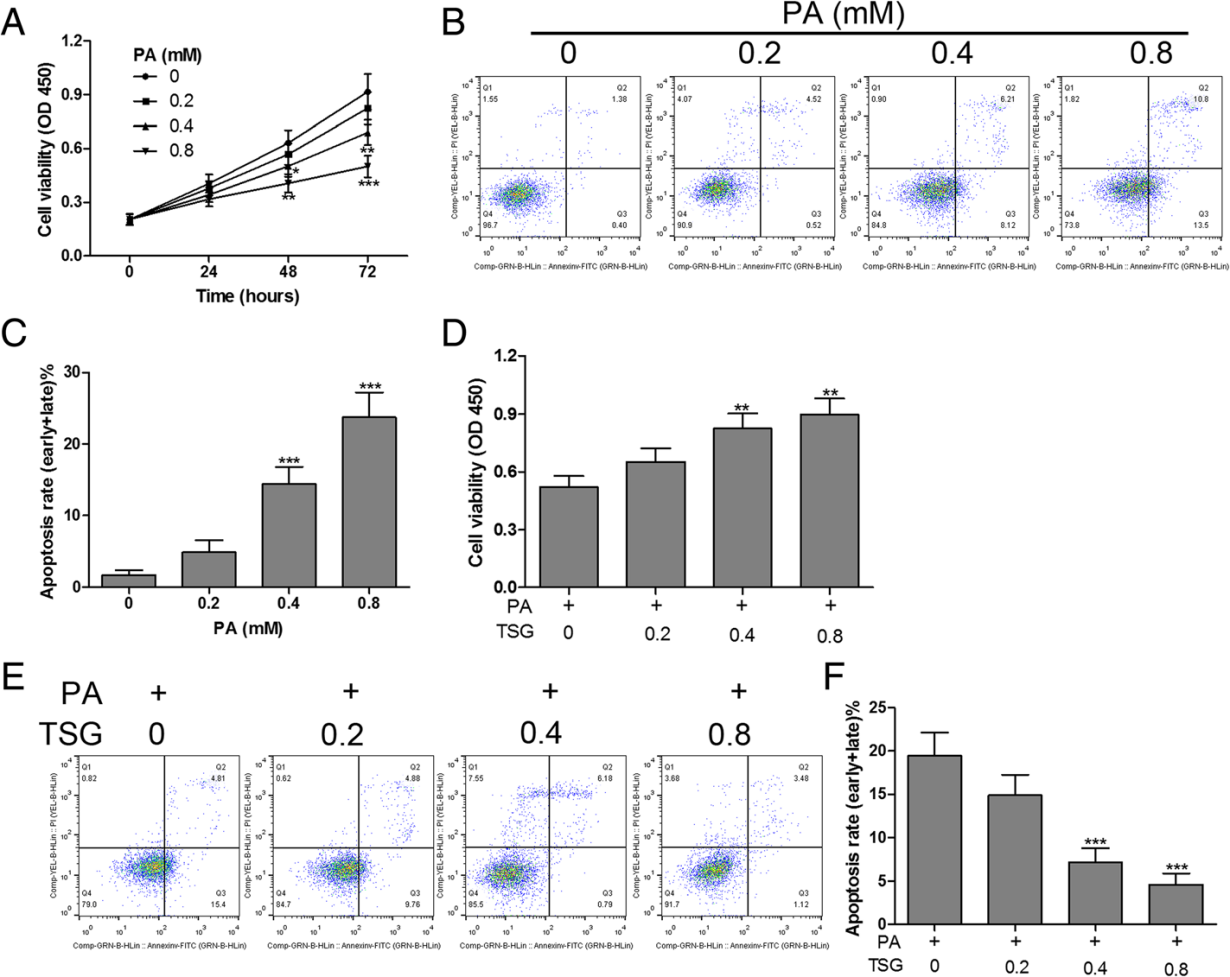
TSG inhibited PA-induced apoptosis in H9c2 cardiomyocytes. After exposure to PA with 0, 0.2, 0.4 and 0.8 mM for different times (0–72 h), cell viability was measured by CCK-8 assay (**a**); cell apoptosis was detected by flow cytometry after incubation with PA (0, 0.2, 0.4 and 0.8 mM) for 48 h (**b** and **c**). Cell viability (**d**) and apoptosis (**e** and **f**) were detected using CCK-8 assay and flow cytometry, respectively, after combined treatment with PA (0.4 mM) and TSG (0–0.8 mM) for 48 h. ^*^ *P* < 0.05, ^**^ *P* < 0.01, ^***^ *P* < 0.001. *n* = 3 in each group

To investigate the effect of TSG on PA-induced cardiomyocyte apoptosis in vitro, we first examined the cytotoxicity of PA in H9c2 cells, which were exposed to PA with different concentrations for 0–72 h. The results demonstrated that H9c2 cell viability was suppressed by PA in a dose- and time-dependent manner (Fig. 1a). Flow cytometry assay revealed that stimulation of H9c2 cells with PA (0–0.8 mM) for 48 h resulted in a significant increase in apoptosis in a dose-dependent manner (Fig. 1b and c). These findings showed that PA induced growth inhibition and apoptosis in H9c2 cardiomyocytes. In addition, we found that PA (0.4 mM) induced growth inhibition and apoptosis in H9c2 cells were relieved by TSG (0.4 and 0.8 mM) treatment for 48 h (Fig. 1d, e and f). These data suggested that TSG exerted a significant cytoprotective effect on PA-induced H9c2 cell injuries.

### TSG attenuated PA-induced inflammatory response in H9c2 cardiomyocytes

**Fig. 2:**
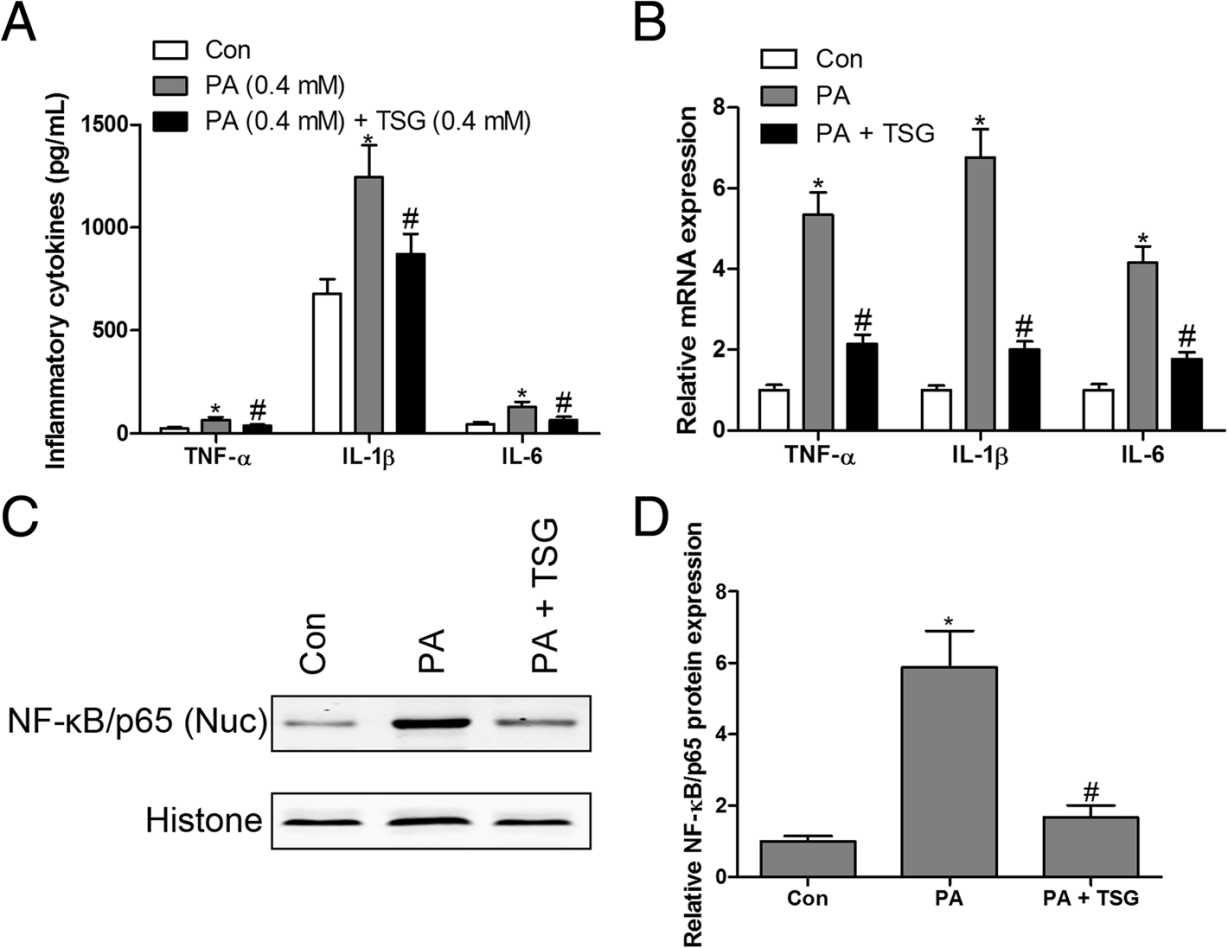
TSG inhibited PA-induced inflammation in H9c2 cardiomyocytes. PA-stimulated H9c2 cells with or without TSG (0.4 mM) for 48 h, TNF-α, IL-1β and IL-6 levels in the supernatant were measured by ELISA kit (**a**); RT-qPCR was performed to measure the mRNA expression of TNF-α, IL-1β and IL-6 (**b**); protein expression of NF-κB/p65 in the nucleus was measured by western blotting (**c** and **d**). ^*^ *P* < 0.05 compared with control group; ^#^ *P* < 0.05 compared with PA group. *n* = 3 in each group

Numerous studies have shown that PA is liable to induce an inflammatory response in a variety of cells [2, 15]. However, the protective effects of TSG on the PA-induced inflammatory response in H9c2 cardiomyocytes remained unknown. To detect the levels of TNF-α, IL-1β and IL-6, H9c2 cells were exposed to PA (0.4 mM) with or without TSG (0.4 mM) treatment. Our results indicated that PA significantly up-regulated the levels of TNF-α, IL-1β and IL-6 compared with the control group, as determined by an ELISA (Fig. 2a) and RT-qPCR assay (Fig. 2b), while TSG treatment abolished the over-activated inflammation of PA in H9c2 cardiomyocytes. In addition, an increased NF-κB/p65 level in the nucleus (Nuc) was detected in PA-treated H9c2 cardiomyocytes, while TSG had the capacity for reduction of the PA-induced up-regulation of NF-κB/p65 protein in the nucleus (Fig. 2c and d). NF-κB as a key transcription factor has been implicated in the PA-induced inflammatory response [16, 17]. Over-activation of NF-κB is associated with cytoplasmic degradation of its inhibitor IκBα, which leads to the translocation of p65, a subunit of NF-κB, into the nucleus, which binds to DNA and enhances the expression of inflammatory cytokines [18]. These results indicated that TSG treatment of H9c2 cells resulted in inhibition of the PA-induced inflammatory response.

### Overexpressed Smad3 neutralized the protective effects of TSG in PA-induced inflammation and apoptosis in cardiomyocytes

**Fig. 3:**
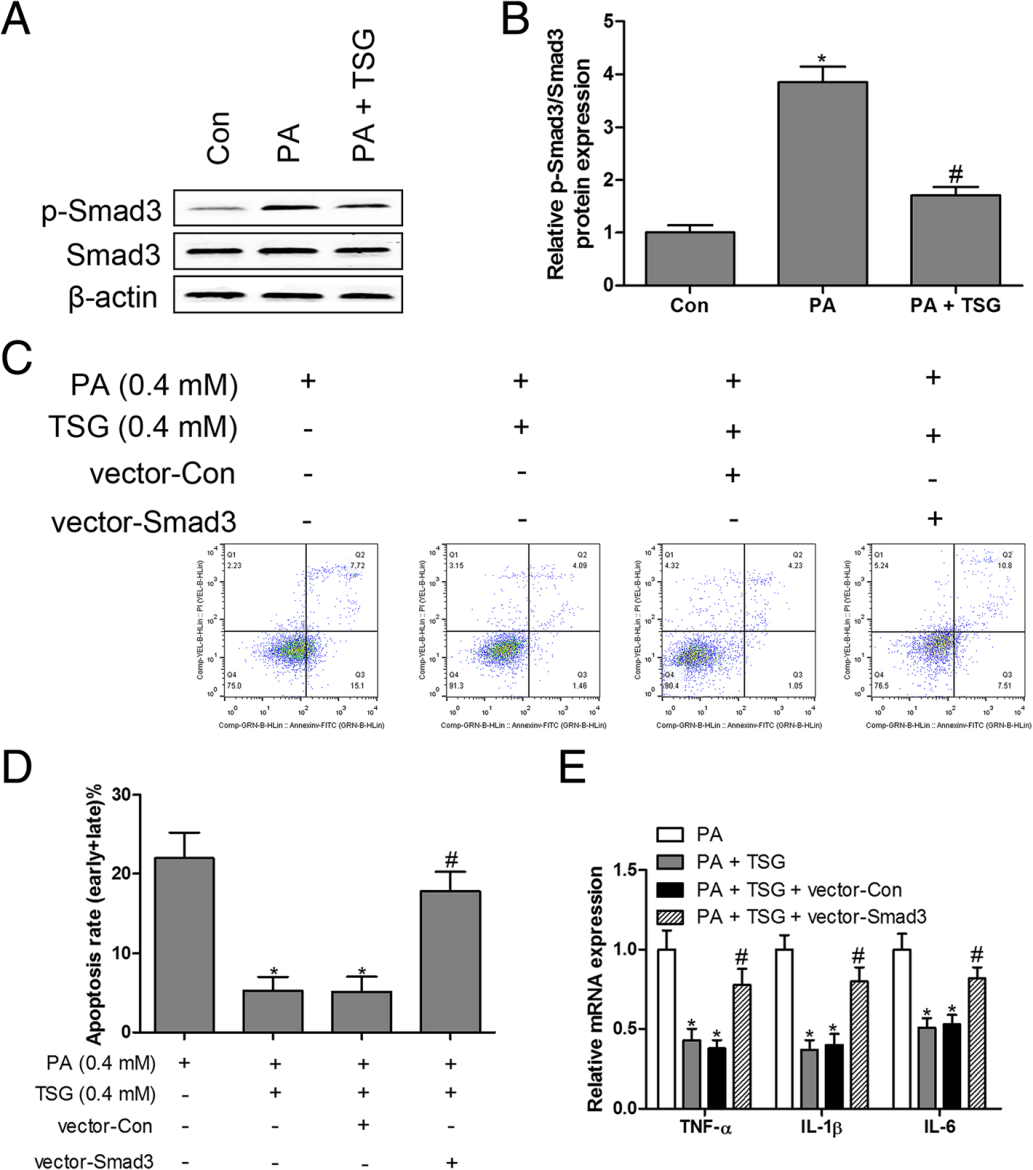
Overexpression of Smad3 neutralized the protective effects of TSG in PA-induced inflammation and apoptosis. Protein expression of Smad3 and p-Smad3 was measured by western blotting (**a** and **b**). H9c2 cells transfected with Smad3 overexpressed plasmids and treated with PA (0.4 mM) and TSG (0.4 mM) for 48 h, cell apoptosis was detected by flow cytometry (**c** and **d**); RT-qPCR was performed to measure the mRNA expression of TNF-α, IL-1β and IL-6 (**e**). ^*^ *P* < 0.05 compared with control group; ^#^ *P* < 0.05 compared with PA or PA + TSG group. n = 3 in each group

To delineate the function of Smad3 in the process of PA-induced inflammation and apoptosis, we first observed the protein expression of p-Smad3 and Smad3 in PA-treated cardiomyocytes, and the data showed that the ratio of p-Smad3 to Smad3 was dramatically elevated in PA-treated cardiomyocytes, whereas TSG reversed the up-regulation of p-Smad3/Smad3 by PA in cardiomyocytes (Fig. 3a and b), suggesting that PA-induced Smad3 phosphorylation might be involved in PA-induced inflammation and apoptosis in cardiomyocytes. To validate this hypothesis, overexpressed Smad3 plasmid was transfected in TSG-treated cardiomyocytes in the presence of PA. The results showed that overexpressed Smad3 eliminated the inhibitory action of TSG in PA-induced apoptosis (Fig. 3c and d) and the inflammatory response (Fig. 3e) in cardiomyocytes. These findings suggested that the phosphorylation of Smad3 as an important regulator mediating PA-induced inflammation and apoptosis in cardiomyocytes.

### TSG suppressed PA-induced inflammation and apoptosis in cardiomyocytes by up-regulating miR-129-3p

**Fig. 4:**
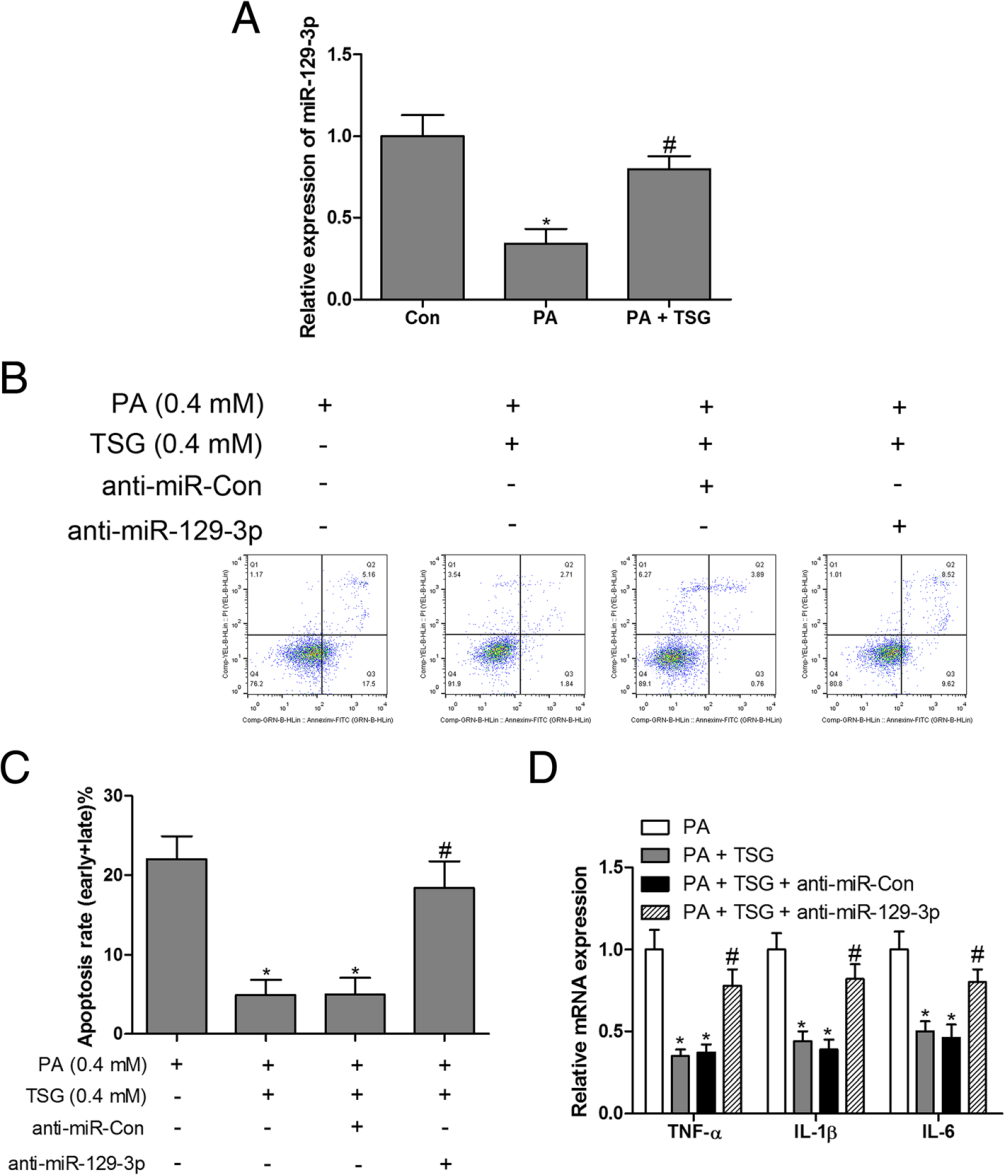
miR-129-3p loss-of-function reversed the protective effects of TSG in PA-induced inflammation and apoptosis. Expression of miR-129-3p was measured by RT-qPCR in H9c2 cells treated with PA (0.4 mM) or PA (0.4 mM) + TSG (0.4 mM) for 48 h (**a**). H9c2 cells transfected with miR-129-3p inhibitors and treated with PA (0.4 mM) and TSG (0.4 mM) for 48 h, cell apoptosis was detected by flow cytometry (**b** and **c**); RT-qPCR was performed to measure the mRNA expression of TNF-α, IL-1β and IL-6 (**d**). ^*^ *P* < 0.05 compared with control group; ^#^ *P* < 0.05 compared with PA or PA + TSG group. n = 3 in each group

To further explore the regulatory mechanism of TSG in PA-induced cardiomyocyte dysfunction, the post-transcriptional mechanism of miR-129-3p was investigated. The results indicated that the expression of miR-129-3p was markedly inhibited by PA compared with the control group, while TSG treatment caused a robust increase in miR-129-3p levels in PA-stimulated cardiomyocytes (Fig. 4a). Subsequently, silencing of miR-129-3p with miR-129-3p inhibitors in PA-treated cardiomyocytes reversed TSG-inhibited apoptosis (Fig. 4b and c) and inflammation (Fig. 4d). These results indicated that TSG treatment of H9c2 cells resulted in inhibition of PA-induced inflammation and apoptosis by up-regulating miR-129-3p expression.

### Smad3 was a direct target of miR-129-3p

**Fig. 5:**
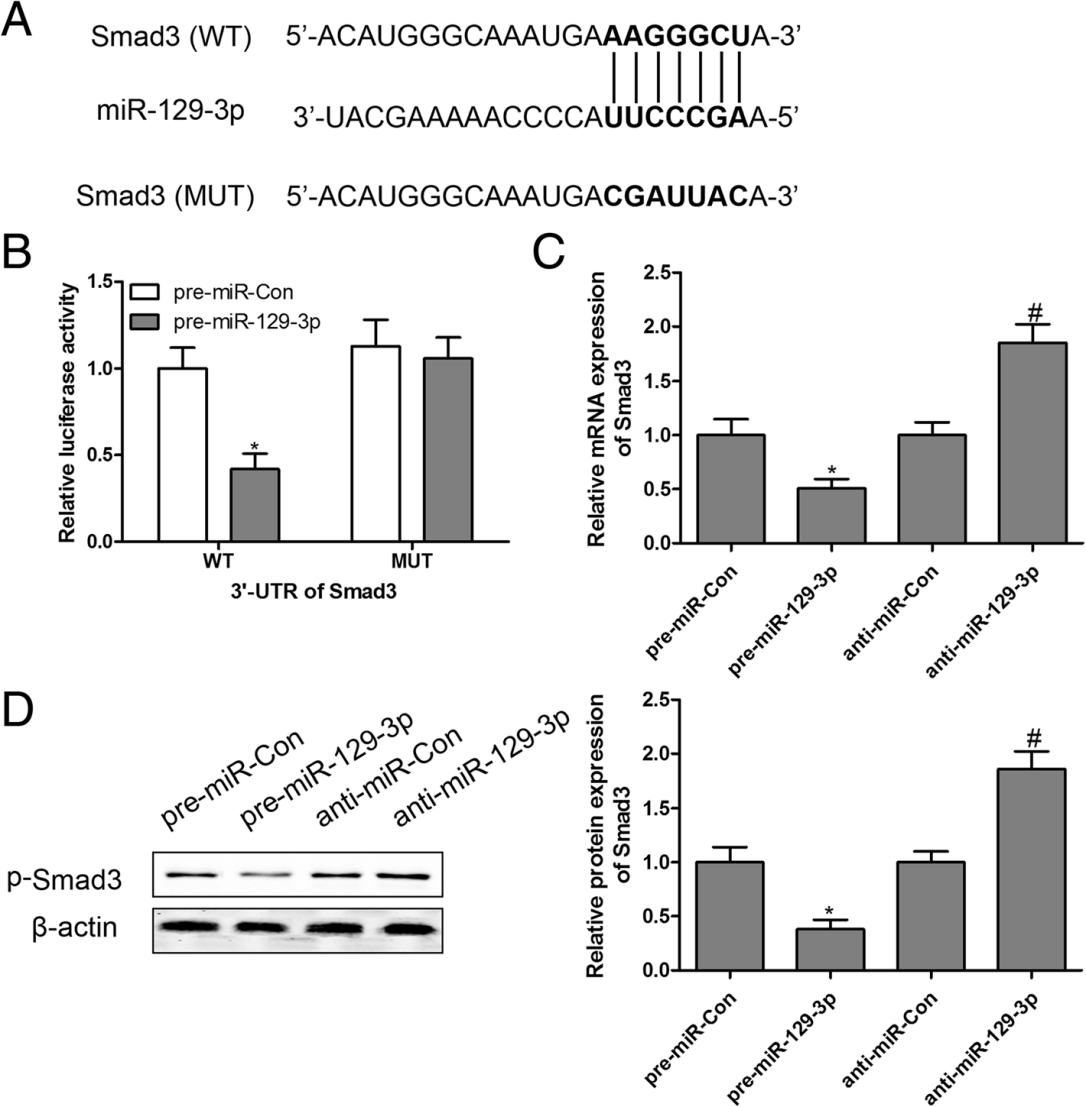
Smad3 is a direct target of miR-129-3p. The putative miR-129-3p binding sites in the 3′-UTR of Smad3 were predicted by on-line software (**a**). The luciferase activity assay was performed (**b**). After transfection with pre-miR-129-3p or anti-miR-129-3p, the mRNA expression of Smad3 (**c**) and protein expression of p-Smad3 (**d**) were assayed by RT-qPCR and western blotting, respectively. ^*^ *P* < 0.05 compared with pre-miR-Con group; ^#^ *P* < 0.05 compared with anti-miR-Con group. n = 3 in each group

Based on above results, we found that TSG treatment decreased the phosphorylation of Smad3 and increased the expression of miR-129-3p, leading to a remarkable improvement of PA-induced H9c2 dysfunction. However, the association between Smad3 and miR-129-3p remains unclear. Using the online prediction software Targetscan (http://www.targetscan.org) and miRDB (http://www.mirdb.org), we found that there was a binding site for miR-129-3p in the 3′-UTR of Smad3 as shown in Fig. 5a. To further elucidate whether miR-129-3p directly targeted Smad3, a luciferase reporter assay was performed in H9c2 cells after transfection into the wild-type (WT) sequence or mutant-type (MUT) sequence of Smad3 combined with pre-miR-Con or pre-miR-129-3p. The results demonstrated that transfection with pre-miR-129-3p significantly decreased the luciferase enzyme activity when co-transfected with WT 3′-UTR of Smad3, but there was no change in luciferase enzyme activity in MUT 3′-UTR of Smad3 (Fig. 5b). We also found that the mRNA expression of Smad3 and protein expression of p-Smad3 were significantly suppressed by pre-miR-129-3p and increased by anti-miR-129-3p in H9c2 cells (Fig. 5C and D). Taken together, these findings suggested that Smad3 was a direct target of miR-129-3p.

### Smad3 showed an antagonistic effect to miR-129-3p in PA-induced inflammation and apoptosis

**Fig. 6:**
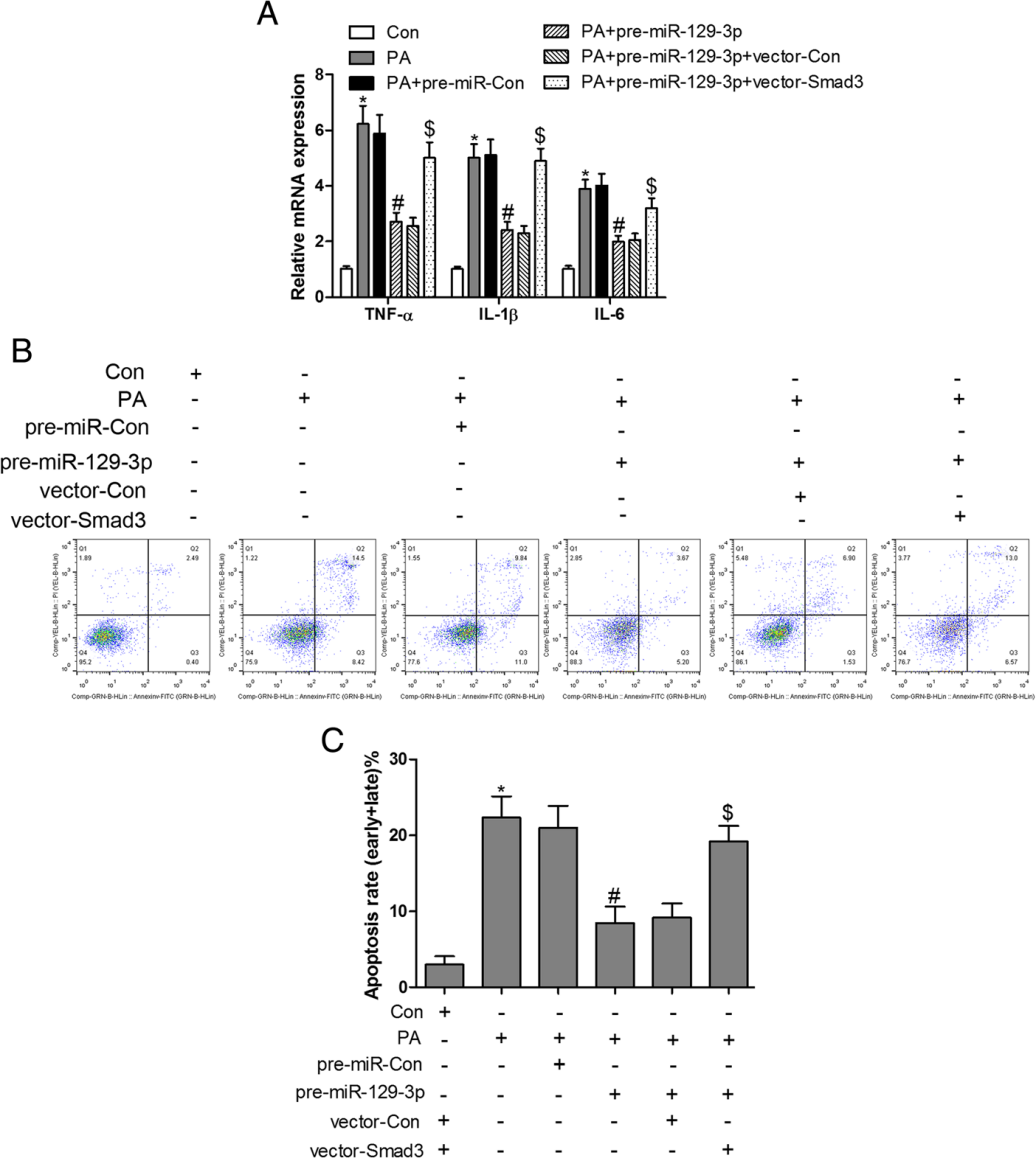
Smad3 shows an antagonistic effect to miR-129-3p in PA-induced inflammation and apoptosis. PA-treated H9c2 cells transfected with Smad3 overexpressed plasmids, pre-miR-129-3p or Smad3 overexpressed plasmids combined with pre-miR-129-3p for 48 h, RT-qPCR was performed to measure the mRNA expression of TNF-α, IL-1β and IL-6 (A); cell apoptosis was detected by flow cytometry (B and C). ^*^ *P* < 0.05 compared with control group; ^#^ *P* < 0.05 compared with PA group; ^$^ *P* < 0.05 compared with PA + pre-miR-129-3p group. n = 3 in each group

To further investigate the interaction between Smad3 and miR-129-3p, overexpressed miR-129-3p and Smad3 were co-expressed in PA-treated cardiomyocytes. Our results confirmed that PA-triggered inflammation and apoptosis could be attenuated by pre-miR-129-3p, while they were abolished by overexpression of Smad3 in H9c2 cells (Fig. 6A, B and C). These observations revealed that miR-129-3p/Smad3 signaling was implicated in PA-induced inflammation and apoptosis and could be regulated by TSG in H9c2 cardiomyocytes.

## Discussion

The present study highlighted that TSG had a beneficial effect on cardiomyocytes from PA-induced inflammation and apoptosis. To our knowledge, TSG could antagonize the PA-mediated cardiomyocytes’ dysfunction by inhibiting p-Smad3 and elevating miR-129-3p expression. Overexpression of Smad3 or miR-129-3p loss-of-function resulted in neutralization of TSG protective effects on PA-stimulated cardiomyocytes. Interestingly, Smad3 was a direct target of miR-129-3p and exerted an antagonistic effect on miR-129-3p that suppressed PA-induced inflammation and apoptosis in cardiomyocytes. Therefore, we summarized that TSG inhibited PA-induced inflammation and apoptosis in cardiomyocytes, and the underlying mechanism was mediated, at least partially, by targeting miR-129-3p/Smad3 signaling.

In our study, we found that PA (400 and 800 μM) activated the inflammatory response, inhibited proliferation and induced apoptosis in H9C2 cells. He et al. revealed that PA (300 μM)-stimulated neonatal rat cardiomyocytes led to a significant increase of cell apoptosis and dysfunction [19]. Li et al. reported that PA (100 μM) induced inflammatory injury and apoptosis in H9C2 cells [2]. Dyntar et al. found that PA (250 and 500 μM) resulted in a significant increase of apoptotic cells in rat adult cardiomyocytes [20]. These results suggested that the concentrations of PA at the same order of magnitude induced cardiomyocyte damage in these studies [2, 19, 20].

Previous studies have confirmed that TSG exerts anti-oxidative stress, anti-inflammation and improved cognitive impairment [7, 21, 22]. The study on the molecular mechanism of TSG in high glucose-induced MPC5 podocyte injury shows that NOD-like receptor family pyrin domain containing 3 (NLRP3) inflammasome-mediated inflammatory response cascade signaling is blocked by TSG [21]. It was noteworthy that the expression of Smad3 was up-regulated in PA-stimulated cardiomyocytes, and overexpression of Smad3 described in the present study neutralized the anti-inflammatory and anti-apoptotic effects of TSG in PA-incubated cardiomyocytes.

Based on previous studies [23, 24], we hypothesized that SMAD3 was involved in PA-induced cell dysfunction. In fact, we found that the expression levels of p-SMAD3 were up-regulated in PA-treated H9C2 cells. It has been reported that Smad3 plays an important role in cardiac pathological processes, such as cardiac fibrosis [25], hypertrophy [26] and heart failure [27]. Smad3 loss-of-function is associated with attenuated cardiomyocyte apoptosis in the remodeling myocardium [28]. The transforming growth factor-β1 (TGF-β1)/Smad3 pathway is activated in hypoxia/reoxygenation and high glucose-induced cardiomyocyte apoptosis [29]. Consistent with these results, our study clearly demonstrated that the phosphorylation levels of Smad3 were up-regulated in response to PA stimulation. TSG treatment inhibited PA-induced apoptosis in cardiomyocytes by decreasing the phosphorylation levels of Smad3. Smad3 as a key transcription factor also mediates the inflammatory response mainly by NF-κB-driven inflammation, which can up-regulate pro-inflammatory cytokines (IL-1β, IL-6 and TNF-α) [30]. Interestingly, the NLRP3 inflammasome can serve as a strong positive feedback loop for activation of TGF-β1/Smad3 signaling modulation of inflammation [31]. In cardiomyocytes, TGF-β1/Smad3 and NF-κB pathways mediate pressure overload-induced apoptosis and inflammation [12]. Our study showed that levels of the pro-inflammatory cytokines TNF-α, IL-1β and IL-6, and NF-κB/p65 were increased in PA-stimulated cardiomyocytes, while TSG administration reversed the PA-triggered inflammatory reaction.

Smad3 associated with apoptosis and inflammation in cardiomyocytes has been confirmed in previous studies [12, 29]. However, comparatively little work has examined the molecular mechanism of miRNAs-targeted Smad3 in PA-induced cardiomyocyte apoptosis and inflammation. To further investigate the post-transcriptional regulatory mechanism, we used on-line prediction software to deduce the potential miRNAs which could target regulation of p-SMAD3 expression. We found that multiple miRNAs (miR-129-3p, miR-23-3p, miR-145-5p and miR-203-3p) could bind to its 3′-UTR. However, preliminary experiments revealed only miR-129-3p in response to PA-treated H9C2 cells. Therefore, we focused on miR-129-3p in our study. A previous study suggested that miR-24 can improve heart function and attenuate fibrosis after myocardial infarction by reducing Smad3 phosphorylation [32]. In our study, we found that Smad3 was a direct target of miR-129-3p, which protected the cardiomyocytes from adverse external stimuli. Jeppesen et al. showed that miR-129-3p may be involved in Ang II-mediated cardiac biology and disease [33]. A genome-wide expression study of circulating microRNAs in patients with heart failure (HF) showed a significant decrease of miR-129-3p in the serum from HF patients [34]. These results suggested that miR-129-3p may be a susceptibility gene and inhibited in the pathological conditions of cardiomyocytes. Our findings provided solid evidence that PA stimulation could suppress miR-129-3p expression and increase Smad3 phosphorylation. Therefore, it is conceivable that the PA-attenuated miR-129-3p signaling might be relevant to the over-activation of inflammation and apoptosis in cardiomyocytes.

However, there were some limitations of our study. We focused on PA-induced H9c2 dysfunction in vitro, while the role of TSG in cardiac function in vivo was not involved. We plan to perform the in vivo experiments in our future research. Moreover, we obtained no direct evidence of an association between NF-κB and miR-129-3p.

## Conclusion

Taken together, the results of our experiment identified up-regulation of p-Smad3 and down-regulation of miR-129-3p expression in PA-stimulated cardiomyocytes. More importantly, we found that TSG protected against PA-induced cardiomyocyte apoptosis and inflammation by stimulating miR-129-3p targeted to inhibit Smad3 signaling. Thus, we speculate that TSG may have a beneficial effect on hyperlipemia-related cardiovascular diseases, which needs to be further studied in some animal models.

## Abbreviations

annexin V-FITC/PI: Annexin V-fluorescein isothiocyanate/polyimide
ELISA: Enzyme-linked immunosorbent assay
HF: Heart failure.
HFD: High-fat diet
IL-1β: Interleukin-1β
IL-6: Interleukin-6
IκBα: NF-κB inhibitor alpha
NF-κB: Nuclear factor-kappa B
NLRP3: NOD-like receptor family pyrin domain containing 3
PA: Palmitic acid
RT-qPCR: Reverse transcription-quantitative polymerase chain reaction
Smad3: Mothers against decapentaplegic homolog 3
TGF-β: Transforming growth factor β
TNF-α: tumor necrosis factor α
TSG: tetrahydroxy stilbene glucoside
